# What is the effect of benzodiazepines on deep brain activity? A study in pediatric patients with dystonia

**DOI:** 10.3389/fneur.2023.1215572

**Published:** 2023-08-11

**Authors:** Estefania Hernandez-Martin, Jessica S. L. Vidmark, Jennifer A. MacLean, Terence D. Sanger

**Affiliations:** ^1^Department of Electrical Engineering and Computer Science, University of California, Irvine, CA, United States; ^2^Department of Biomedical Engineering, University of California, Irvine, CA, United States; ^3^Department of Neurology, Children’s Health Orange County (CHOC), Orange, CA, United States

**Keywords:** benzodiazepines, deep brain stimulation, dystonia, evoked potential, frequency analysis

## Abstract

**Introduction:**

Benzodiazepines (BDZs) are commonly used to treat the symptoms of movement disorders; however, deep brain stimulation (DBS) has become a popular treatment for these disorders. Previous studies have investigated the effects of BDZ on cortical activity, no data are currently available on their effects on deep brain regions, nor on these regions’ responses to DBS. How the BDZ affects the thalamus and basal ganglia in dystonia patients remains unknown.

**Methods:**

DBS recordings were performed in ventral oralis anterior/posterior (VoaVop), ventral intermediate (VIM) and ventral anterior (VA) thalamic subnuclei, as well as globus pallidus interna (GPi) and subthalamic nucleus (STN). Evoked potentials (EP) and frequency domain analysis were performed to determine the BDZ effect on neural activities compared to the control condition (off-BDZ). Three male pediatric patients with dystonia treated with BDZ and undergoing depth electrode evaluation for clinical targeting were recruited for the study. Stimulation was administered at 25 and 55 Hz frequencies and recordings were simultaneously gathered through pairs of externalized stereoelectroencephalography (sEEG) electrodes. EP amplitude and the effect of stimulation on the frequency spectrum of activity were compared at baseline and following clinical administration of BDZ.

**Results:**

Frequency analysis showed consistent reductions in activity during BDZ treatment in all studied brain regions for all patients. Evoked potential (EP) analysis showed increased subthalamic nucleus (STN) EP amplitude and decreased ventral intermediate (VIM) and STN EP amplitude during BDZ treatment.

**Interpretation:**

BDZs reduce thalamic and basal ganglia activity in multiple regions and alter the efficacy of transmission between these regions. While the mechanism is unknown our results confirm the known widespread effects of this class of medications and identify specific areas within the motor system that are directly affected.

## 1. Introduction

Dystonia is defined as a movement disorder in which involuntary sustained or intermittent muscle contractions cause twisting and repetitive movements, abnormal postures, or both (1). The mechanism underlying childhood dystonia is not fully understood but may include abnormal patterns of subcortical activity, excessive basal ganglia or peripheral loop gain, or decreased focusing of intended patterns of muscle activity (2, 3). Hyperkinetic movement disorders are a group of neurological disorders that involve abnormal involuntary movements, such as chorea, athetosis, ballismus, myoclonus, and tics, among others. These disorders can be caused by a variety of factors, including genetic mutations, medication side effects, brain injury, and neurodegenerative conditions (4). The treatment of dystonia and other hyperkinetic movement disorders is complex and individualized. It may involve physical therapy, botulinum toxin injections, deep brain stimulation or medications such as benzodiazepines (BDZs) (5). BDZs are a commonly used class of medication frequently used in the treatment of hyperkinetic movement disorders, including the hyperkinetic components of dystonia (4).

BDZs act as direct gamma-aminobutyric acid (GABA) receptor agonists, whose effect is at least partially mediated by GABA receptor activation (6). They bind to and activate GABA receptors, which are the main inhibitory neurotransmitter receptors in the brain, and their activation results in a reduction in neuronal excitability and anxiolytic, sedative, hypnotic, and anticonvulsant effects (7). BDZs modulate the activity of GABAergic neurons through the link to two types of GABA receptors, GABAa and GABAb (8). Overall, BDZs affect the background spontaneous firing and the neuronal spike activity patterns of basal ganglia nuclei (9), mainly through activation of GABA that is the major inhibitory neurotransmitter within the basal ganglia (10). GABA receptors are also present in the thalamus, which mainly contains GABAb receptors that play a crucial role in the regulation of sensory information processing (11). The activation of GABAb receptors in the thalamus inhibits the release of excitatory neurotransmitters, which reduces the excitability of thalamic neurons and therefore the transmission of information to the cortex (12). In both human and animal studies, it has been shown that enhancement of GABAergic input alters the level and pattern of firing activity of pallidal neurons in normal and pathological conditions (13).

Nevertheless, the action mechanisms of BDZs are not understood, but they seem to act at pharmacologically specific binding sites by linking to the GABA receptors. While many studies have investigated the effects of BDZs on cortical activity, such as increased beta band power (14–16), no data are currently available on their effects on globus pallidus interna (GPi), subthalamic nucleus (STN) and thalamus. One way to study the BDZ effects on deep brain activity is through recordings collected from temporary stereoelectroencephalography (sEEG) leads used during clinical evaluation for deep brain stimulation (DBS) targeting (17, 18).

Based on the GABAergic projections in the basal ganglia, it is plausible that the BDZs would link to GABA receptors contained in the pallidum which are more abundant than in the thalamus (19). However, based on the heterogeneous distribution of the GABA receptors across the basal ganglia and thalamus, it is likely that BDZs affect the entire motor pathway, regardless of whether the conditions are normal or pathological.

In order to study the effects of BDZ on the activity of multiple deep brain structures in three awake dystonic patients while they were at rest, we performed a retrospective analysis of the basal ganglia nuclei (STN and GPi) and the following thalamic nuclei: ventral oralis anterior/posterior (Voa/Vop), ventral intermediate (VIM), and ventral anterior (VA) activity while the patients were undergoing BDZ treatment and when they were off BDZ as part of their clinical assessment period.

## 2. Material and methods

### 2.1. Patients and study design

Retrospective analysis of data of three pediatric patients undergoing a staged procedure to determine optimal areas for implantation of DBS leads for the treatment of dystonia were utilized in this case study (Table 1). The diagnosis of dystonia was established by a pediatric movement disorder specialist (TS) using standard criteria (20). Patients had previously failed conventional medical therapy, and the patients and parents provided signed informed consent for the surgical procedures in accordance with standard hospital practice. The patient and parents also consented, or assented as applicable, to the research use of electrophysiological data collected during the clinical procedure, as well as a Health Insurance Portability and Accountability Act (HIPAA) authorization for the research use of protected health information.

**Table 1:** Demographic characteristics of the patients included in the benzodiazepines (BDZ) study.

Patient	Etiology	Characteristics	Gender	Age (yrs)	Implanted regions (bilateral)	On-BDZ sEEG times	Benzodiazepine doses
1	Glutaric aciduria type I	Hypertonic dystonia	M	13	GPi, STN, VA, VIM, Voa/Vop	Day A: 12–1 pm Day B: 4 pm Day C: 10:30 am	Day A: 8:43 am and 12:08 (2 mg diazepam, IV) Day B: 2:34 pm (1.5 mg diazepam, IV) Day C: 9:08 am (1.5 mg diazepam, IV)
2	KMT2B (genetic)	Chorea, dystonia	M	10	GPi, STN, VA, VIM, Voa/Vop, PPN	3–4:30 pm	6:44 am (3 mg diazepam, enteral)
3	Perinatal hypoxic-ischemic injury	Tremor, dystonia	M	14	GPi, STN, VA, VIM, Voa/Vop, PPN	6 pm	3:20 pm, 4:32 pm (0.5 mg clonazepam, enteral)

M, male; IV, intravenous; GPi, globus pallidus interna; STN, subthalamic nucleus; VA, ventral anterior; VIM, ventral intermediate; Voa/Vop, ventral oralis anterior/posterior; PPN, pedunculopontine nucleus, IV, intravenous.

All data were recorded during two intervals of 5 min in the neuromodulation monitoring unit (NMU) (18). For most patients, the latter interval was the control condition, corresponding to a resting period without benzodiazepine treatment (off-BDZ) after weaning off benzodiazepines to allow for better assessment of response to stimulation clinically. All patients had not received any benzodiazepines within 24 h of the off-BDZ period recording. The other interval was the on-BDZ condition, corresponding to a resting period after the patient had been treated with benzodiazepines (Table 1).

### 2.2. Surgical procedure

Our standard clinical procedure for determining DBS targets includes the implantation of 6–10 temporary stereoelectroencephalography (sEEG) depth electrodes (MM16C, Adtech Medical Instrument Corp., Oak Creek, WI, United States) at potential DBS targets (including basal ganglia and thalamic nuclei), as identified based on clinical symptomatology and imaging in each patient (18). Depth electrodes were implanted bilaterally into typical target regions for children with dystonia, which involve thalamic subnuclei such as VA, Voa/Vop, and VIM, and basal ganglia, such as STN and GPi.

The depth electrodes were placed using standard stereotactic procedure, with the most distal stimulation contact placed at the deepest target location. Based on the configuration of these electrodes, the distal contacts of one lead per hemisphere would be in STN, with the proximal contacts in the Voa/Vop area of thalamus. Electrode location was confirmed by co-registration of the preoperative T1-weighted and postoperative computed tomography (CT) scans. Thalamic targeting was also confirmed by identification of leads in subnuclei known to have greater or lesser response to median nerve electrical stimulation (21).

### 2.3. Electrophysiological recordings

Recordings were performed during the one to two weeks following clinical implantation of the temporary depth leads. Each electrode lead has a diameter of 1.2 mm and contains 6 low-impedance (1–5 kΩ) ring macro-electrodes with 2 mm height and 5 mm spacing, as well as 10 high-impedance (70–90 kΩ) micro-electrodes (50 μm diameter). The microelectrodes are arranged in groups of 2 or 3, spaced evenly around the circumference of the electrode shaft, between pairs of ring electrodes. The electrodes were connected to connectors (Cabrio™, Tucker-Davis Technologies Inc., Alachua, FL, United States) that were modified to include a custom unity-gain preamplifier for each microelectrode to reduce noise and motion artifacts. Macroelectrodes bypass the preamplifiers in order to allow for external electrical stimulation. All data reported here are from the high-impedance microelectrode recordings. Microelectrode signals were amplified, sampled, and digitized by a TDT PZ5M analog-to-digital amplifier connected to an RZ2 digital signal processor. Data were streamed to an RS4 high-speed data storage unit, controlled by recording software (System3, TDT). The microelectrode signals were recorded from all implanted electrodes, sampled at 24 kHz, and stored for offline analysis.

### 2.4. Stimulation protocol

The constant intracranial testing consisted of stimulations of 90 μs, 3 V charge-balanced pulses at frequencies of 25 and 55 Hz, administered unilaterally through two adjacent macro-contacts (anode and cathode). Approximately 1,200 repetitions of stimulation were administered per stimulation frequency.

**Figure 1:**
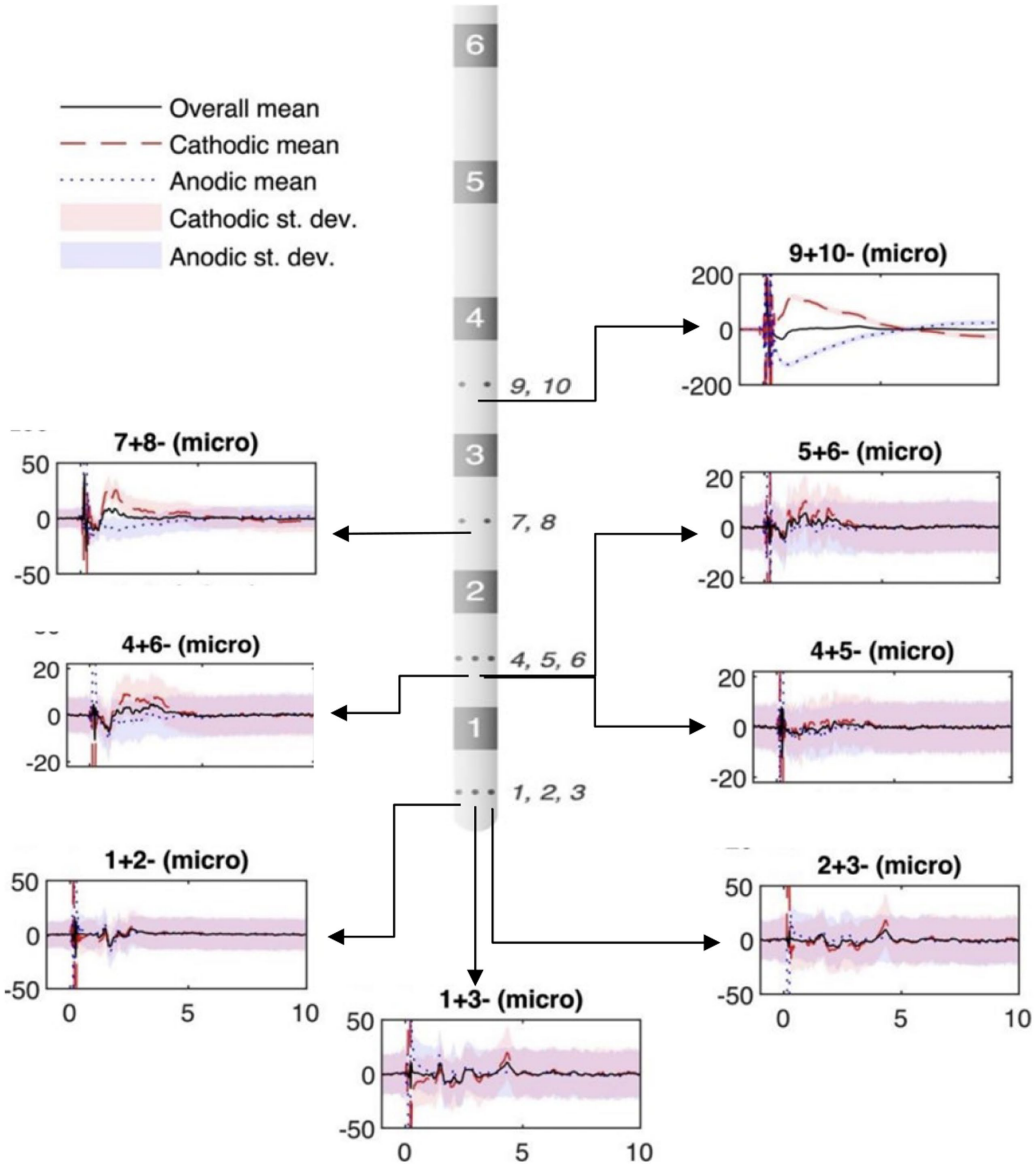
Example of micro-contact in ventral intermediate (VIM) during subthalamic nucleus (STN) stimulation. The stereoelectroencephalography (sEEG) lead diagram displays bipolar recordings from high-impedance micro-contacts (small circles on sEEG lead, numbered to the side). The location of the plots in the figure relate to the recording location with respect to the sEEG lead diagram. Black solid lines: polarity-reversed average; red dashed lines: cathodic stimulation average; blue dotted lines: cathodic stimulation average; shadings: standard deviation. Vertical axis depicts voltage (μV). Horizontal axis shows the time (ms).

For artifact cancellation purposes, both “cathodic” and “anodic” stimulations recordings of intracranial stimulation were utilized. That is, the two stimulation contacts (“1” and “2”) were stimulated as 1 + 2-(1 = anode; 2 = cathode), and then 2 + 1-(2 = anode, 1 = cathode), with the results averaged in the subsequent data analysis. This polarity reversal technique takes advantage of the nonlinearity of the neural response by inverting the polarity of the stimulation artifact, while preserving the polarity of the evoked potential. Therefore, the inverted cathodic and anodic stimulation artifacts not only help distinguish artifacts from neural responses, but also cancel each other out to reduce artifact contamination in the average response (Figure 1).

### 2.5. Data analysis

#### 2.5.1. Power spectral density

All recordings were processed in Matlab (Matlab 2021a). The unavoidable realities of mechanical, physical, and thermal noise decrease the signal-to-noise ratio (SNR), making it difficult to detect neural activity. Therefore, we used orthogonal multilevel wavelet decomposition (MWD) (22) (Matlab function “MODWT”) for signal filtering prior to power spectral density (PSD) analysis. Before analysis, data portions that held obvious electrical artifacts were rejected, based on visual inspection. Data recorded from those microcontacts placed within deep brain targets (visually confirmed through neuroimaging) were used for the analysis in the frequency domain, using fast Fourier transform based methods (Matlab function “fft”). The power spectra amplitudes were normalized with respect to their standard deviations (*z*-score).

Finally, power spectrum density grand averages were taken of the 300 s of recordings across all microcontacts within brain targets, in both conditions (on-BDZ and off-BDZ). The power spectrum was calculated for a 300 s segment in each contact. Then, the power spectra from all contacts on an electrode were combined to determine the average power spectrum for each electrode. Finally, the average power spectra for each electrode in one condition were compared to the average power spectra for the same electrodes in another condition. The nonparametric Wilcoxon signed-rank test was used to compare the spectral features between both conditions. The significance threshold was set at 0.05 with Bonferroni correction for multiple comparisons.

#### 2.5.2. Evoked potential and connectivity analysis

The recorded neural activity was defined as the relative response between two nearby micro-contacts (i.e., local bipolar recordings) and was time-averaged, time-locked to the time of the stimulus artifact. For both conditions, on-BDZ and off-BDZ, the raw data were upsampled, searched for stimulus artifacts, and split into 11 ms segments. All ~1,000 segments not flagged as outliers were then aligned to each other through cross-correlation of an artifact, defined as time “0,” and finally averaged. This stimulus-triggered averaging procedure greatly increased the signal-to-noise ratio (SNR) of the final average response. A double exponential model was implemented to fit to and remove any decay artifacts that could skew the detection and characterization of evoked potentials (EPs) (23). The above process was repeated for both polarity-reversed stimulation types (cathodic and anodic), the results of which were finally averaged to obtain an artifact-reduced neural response.

A peak analysis (matlab function “findpeaks”) was conducted to investigate the consistency of EP peaks across individual recordings. The peaks found in each of the 1,000 individual stimulation segments were identified by setting a minimum prominence threshold equal to half the standard deviation of each segment. Indices where peaks were detected frequently, which occurred more often than twice the standard deviation of the peak occurrences across all indices, were recorded and compared to the peaks of the stimulus-averaged response. When an EP was detected in both conditions, on-BDZ and off-BDZ, the sample pair of EP peak-to-peak (P2P) values was included in the final statistical comparison of EP amplitudes between the two conditions. Non-normal difference distributions used the Wilcoxon signed rank test, while normal difference distributions were analyzed using the paired *t*-test. The significance threshold was set to 0.05.

## 3. Results

Both the power spectra and the EPs from 25 and 55 Hz DBS show differences between the two conditions (on-BDZ vs. off-BDZ) throughout multiple deep brain regions in the thalamus and basal ganglia.

### 3.1. Individual frequency analysis

In order to identify the BDZ effects on the micro-LFP frequency bands, based on the PSDs, a frequency band analysis was calculated to compare the differences between both conditions for each recorded cerebral side (Supplementary Figure S1). A marked reduction of the power in the benzodiazepine condition (on-BDZ) compared to baseline (off-BDZ), ^*^p < 0.05, was seen in all targeted deep brain structures in all three patients. The power spectra show the grand average across all microelectrode recordings for basal ganglia and thalamic nuclei recordings for both conditions. PSDs depict brain signals from the GPi, VA, Voa/Vop, VIM and STN within the frequency band of 1 to 50 Hz. Recordings over 50 Hz did not show any significant differences between both conditions (hence, they are omitted from these visualizations).

**Figure 2:**
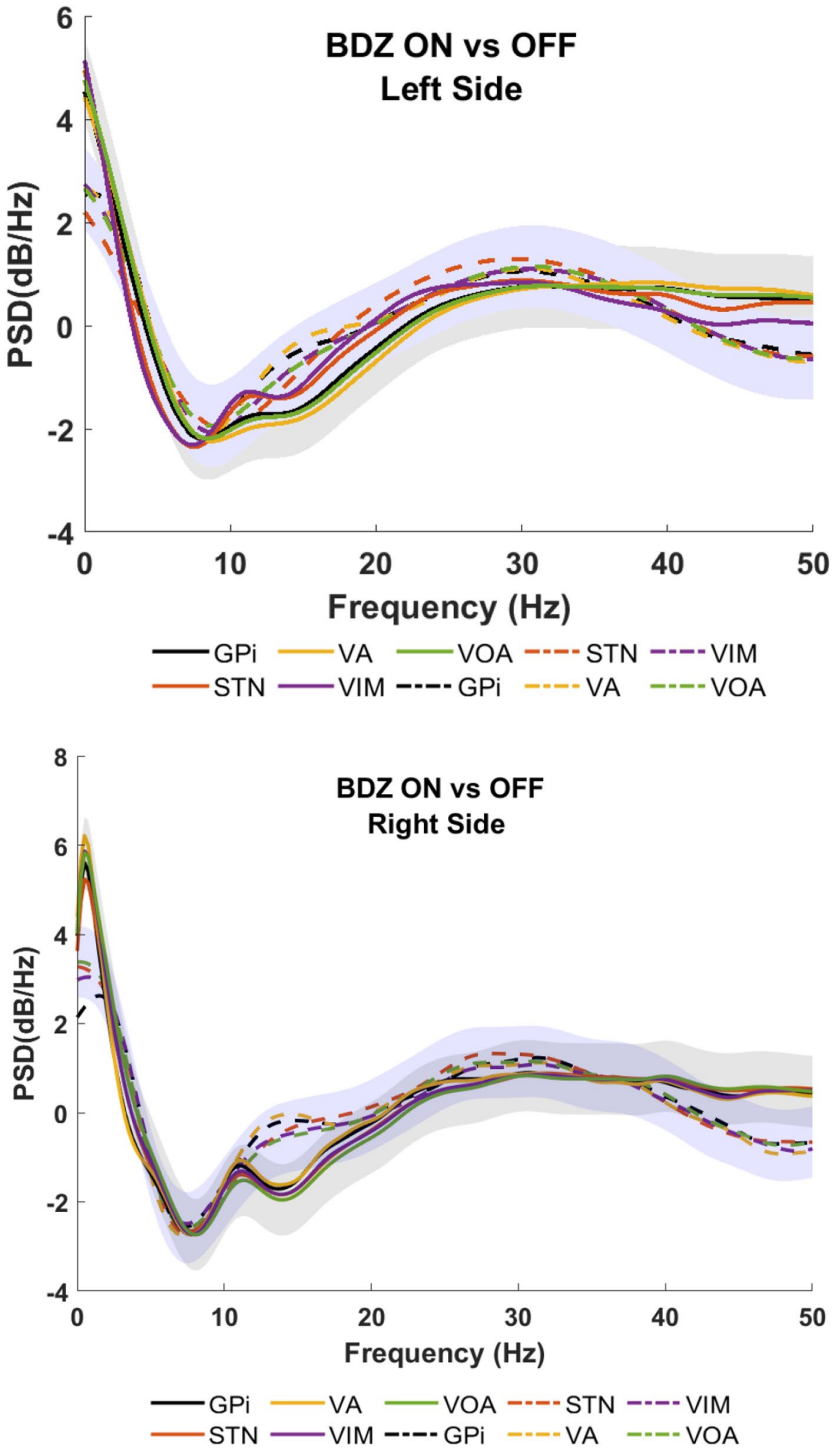
Power spectral analyses (PSDs) for basal ganglia and thalamic subnuclei while the patient is on benzodiazepine (continuous lines) versus the control condition (dashed lines) for patient 1. Top image shows the left hemisphere, and the bottom image shows the right hemisphere. The PSDs show the grand average (lines) and standard deviation (shaded) for all microelectrodes placed bilaterally in GPi (black), STN (orange), VA (yellow), VIM (purple), and Voa/Vop (green). Vertical axis: power (dB/Hz). Horizontal axis: frequency (Hz).

Figure 2 shows the PSDs for patient 1 undergoing DBS implanted on bilateral GPi, STN, VA, VIM and Voa/Vop. Recordings show a reduction of the amplitude during the on-BDZ condition compared to control (off-BDZ). The greatest power drop is around 10–20 Hz, while the power recovers above 20 Hz. This is true for both conditions, although the on-BDZ condition contains less power (amplitude) than the off-BDZ condition, for both hemispheres, and for all deep brain structures.

**Figure 3:**
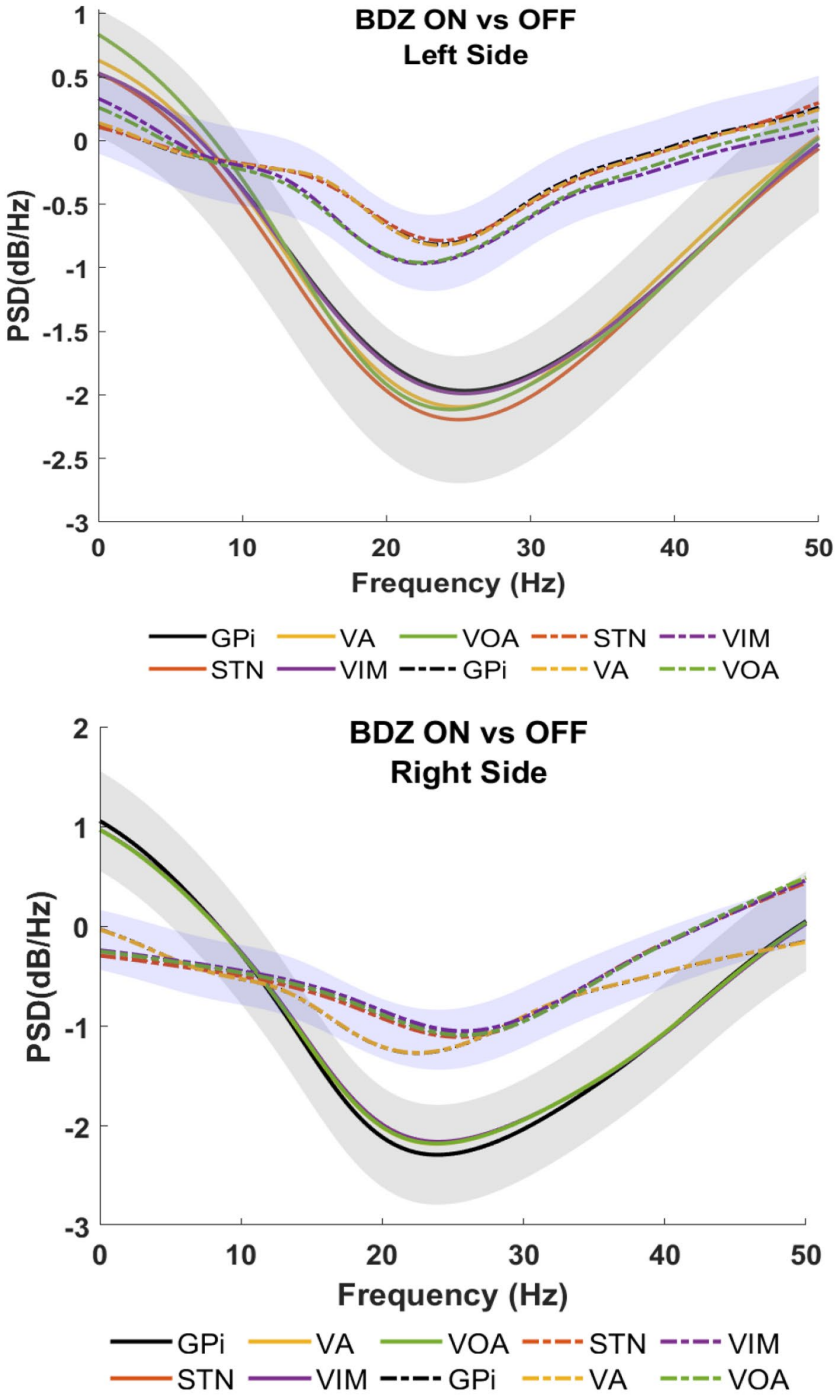
Power spectral analyses (PSDs) for basal ganglia and thalamic nuclei while the patient is on benzodiazepine (continuous lines) versus the control condition (dashed lines) for patient 2.

Figure 3 shows patient 2 with a strong reduction of the power in on-BDZ compared to control (off-BDZ). In contrast to patient 1, all deep brain structures in patient 2 are decreased with the largest power drop for all targeted brain regions, which is around 25 Hz.

**Figure 4:**
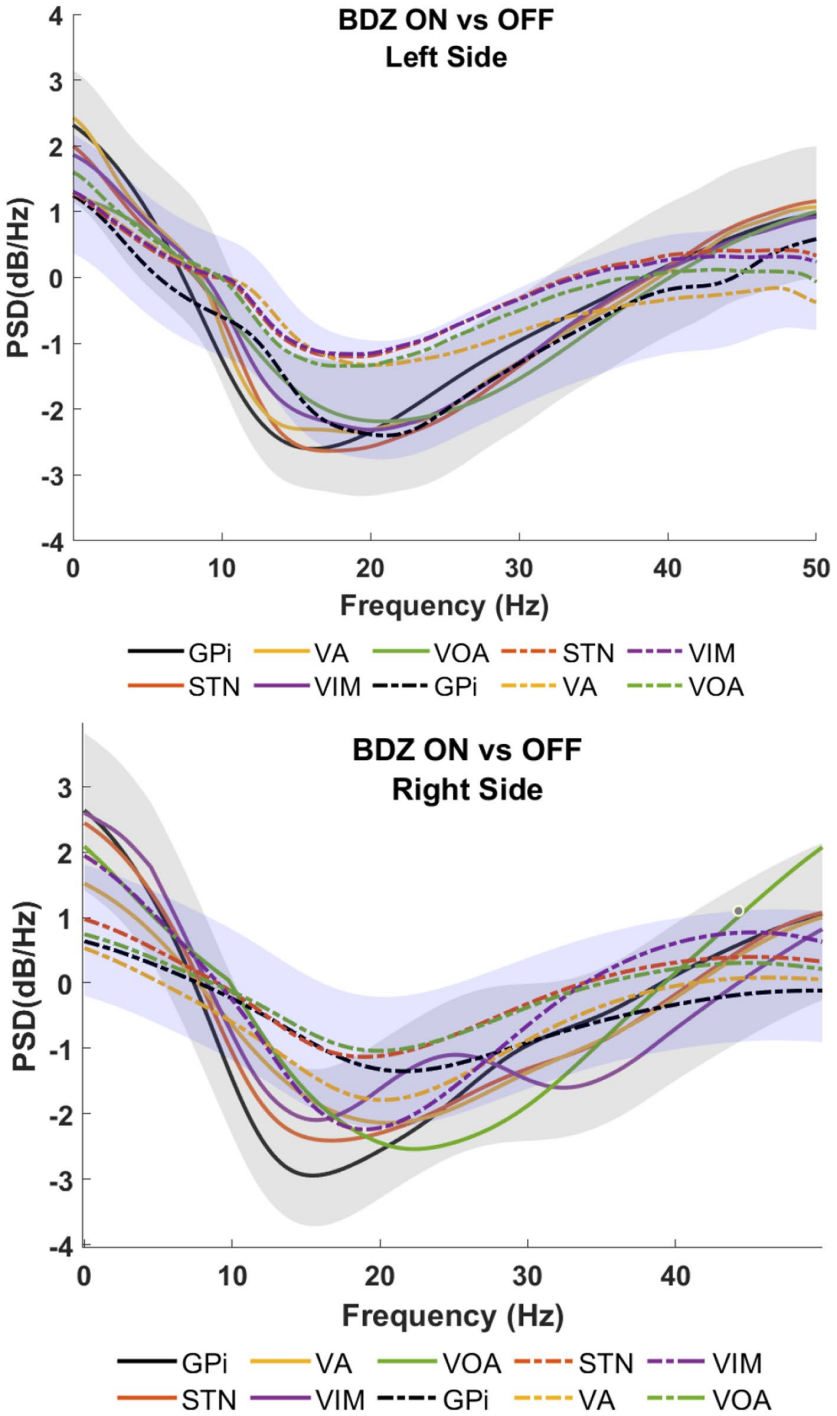
Power spectral analyses (PSDs) for basal ganglia and thalamic subnuclei while the patient is on benzodiazepine (continuous lines) versus the control condition (dashed lines) for patient 3.

Patient 3 also exhibited a reduction in power, with the largest drop observed around 25 Hz (as shown in Figure 4), which is in contrast to the findings of patients 1 and 2. In patient 1, significant reductions in power are observed in the STN during the on-BDZ condition. In patient 2, a strong reduction in power is seen on both sides for all targets. In the case of patient 3, the reductions are most prominent (*p* < 0.05) in the GPi, VA and Voa/Vop for both cerebral sides (as indicated in Supplementary Figure S1).

### 3.2. Group frequency analysis

**Figure 5:**
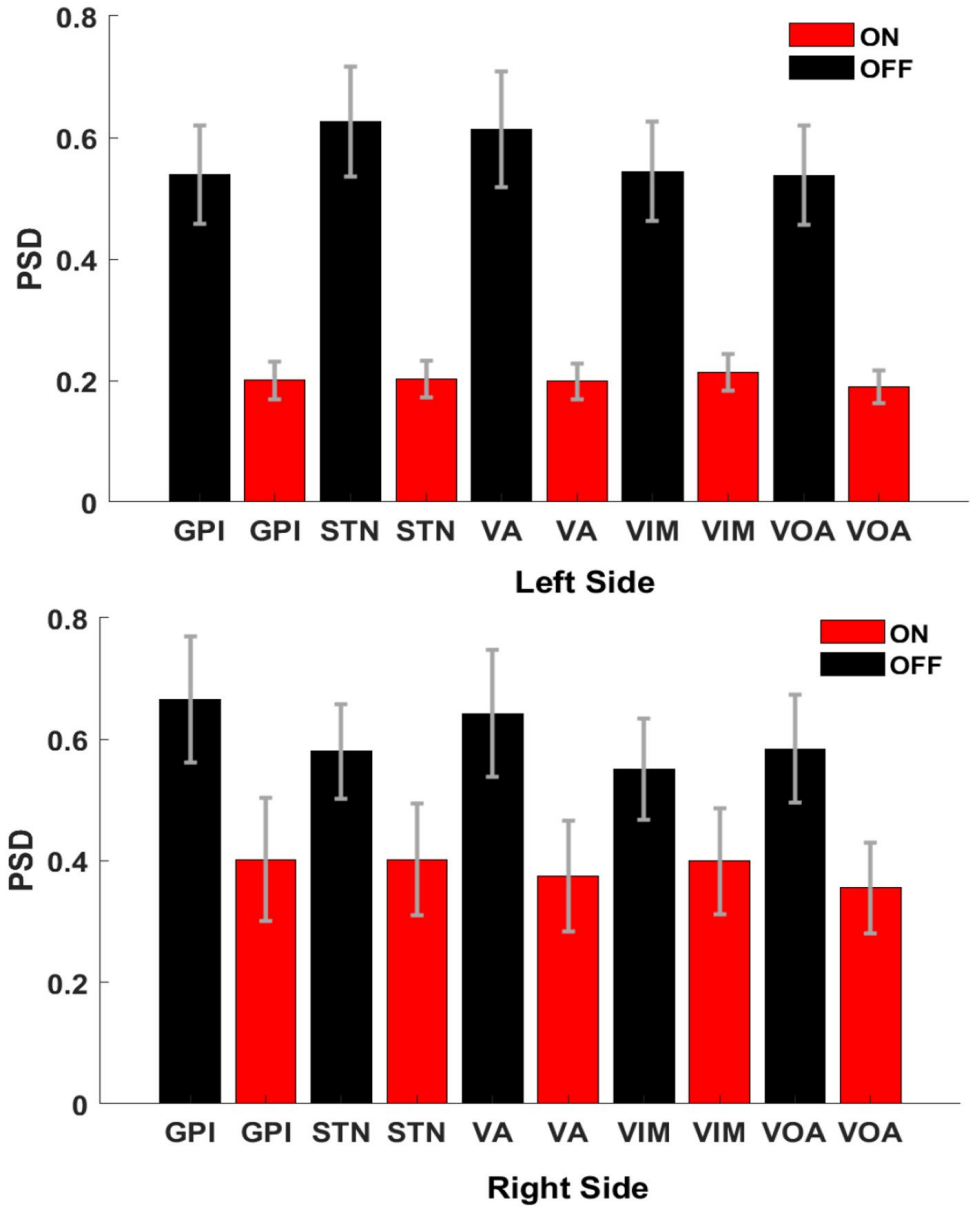
Bar graphs showing the averages and standard deviations of the PSD amplitudes between both conditions, on benzodiazepine (red bars) versus off benzodiazepine (black bars), for left hemisphere (top image) and right hemisphere (bottom image). There are significant differences between both conditions (*p* < 0.05) for all targeted deep brain regions across all patients (*n* = 3). Vertical axis: normalized power difference (dB/Hz). Horizontal axis: targeted deep brain structure.

In order to show the global power reduction across the patients, a group analysis of the PSDs (1–50 Hz) was calculated for each cerebral side. Figure 5 clearly shows the amplitude differences between off-BDZ (black bars) and on-BDZ (red bars); *p* < 0.05 across all patients for both hemispheres. The BDZ effects can be clearly seen on the activity of basal ganglia and thalamus nuclei with a strong reduction in all targeted deep brain regions. The reduction is greater (relative differences ~0.4) in the left side than the reduction (relative differences ~0.2) in the right side.

### 3.3. Benzodiazepine effects on deep brain stimulation evoked potentials

**Figure 6:**
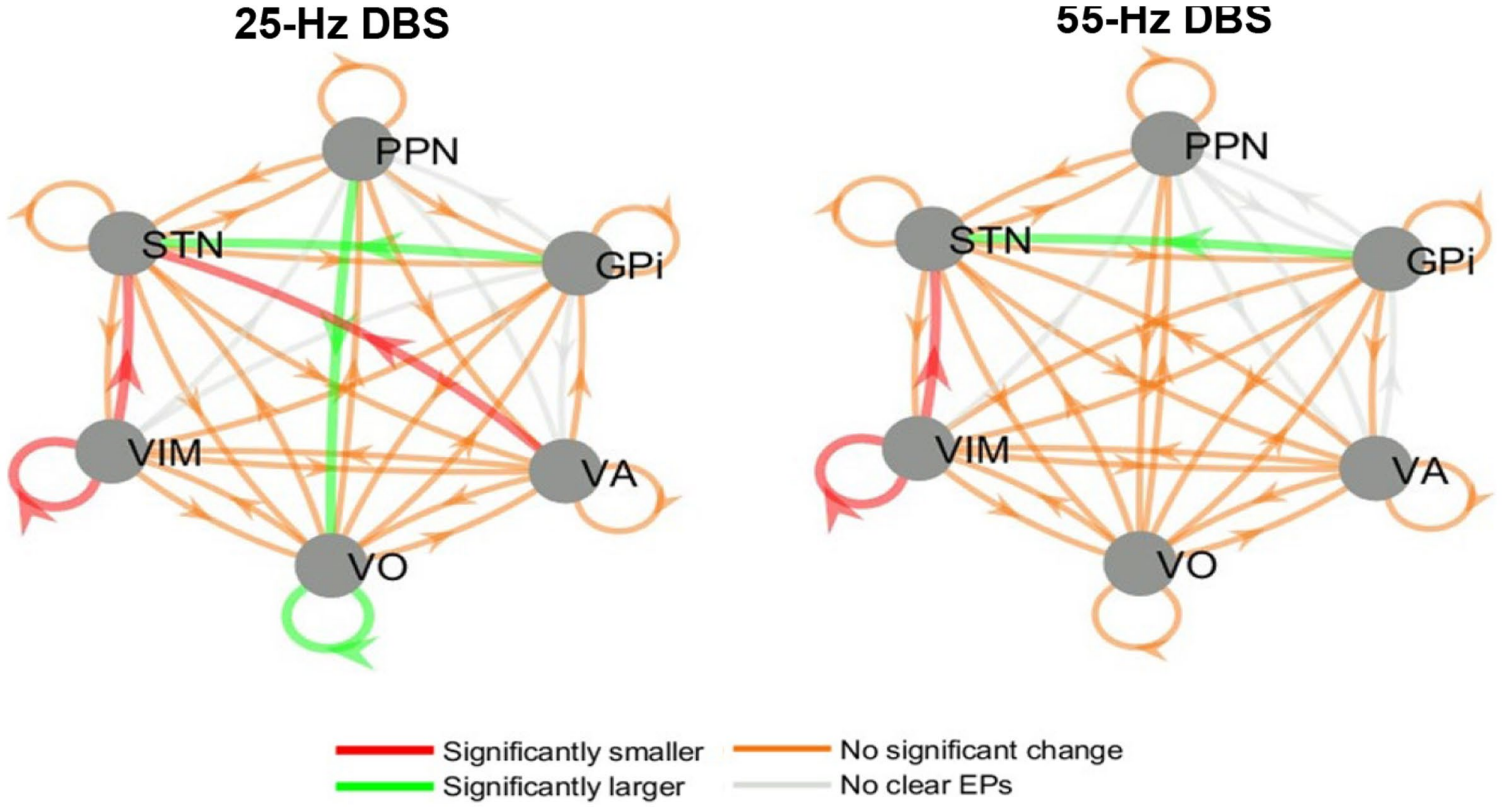
Directed graphs showing the group effects of benzodiazepines on EP amplitude during 25 and 55 Hz stimulation. Left: 25 Hz DBS, right: 55 Hz DBS. Results are from the group analysis of all three patients. The arrows point from each nucleus where DBS was delivered to each nucleus where recordings were available (and an EP may have been elicited). Red/green arrows denote connections where the amplitudes of the EPs significantly decreased/increased when benzodiazepines were administered. Orange/gray arrows symbolize connections where EP amplitudes were not significantly affected/no clear EPs were seen.

Evoked potentials were measured across all deep brain structures while stimulations were applied at 25 and 55 Hz (separately). All evoked potentials were analyzed using their peak-to-peak amplitude and then compared between conditions (on-BDZ versus off-BDZ), with the results represented as connectivity graphs in Figure 6. In two patients, the distal contact of the VIM lead in each hemisphere was targeted into pedunculopontine nucleus (PPN). While the pedunculopontine nucleus (PPN) is a complex structure located in the brainstem and is known to be involved in motor control via the corticospinal pathway, our study focused specifically on the basal ganglia-thalamus pathways. Therefore, we did not use data from the PPN for our analysis.

While some variability was seen in the DBS EP amplitude results between stimulation frequencies, patients and hemispheres, there were also common trends between the whole-brain group analyses at 25 and 55 Hz. These common themes include larger STN EPs during GPi DBS and smaller VIM EPs and STN EPs during VIM DBS. In addition, 25 Hz (but not 55 Hz) DBS elicited larger EPs in Voa/Vop during Voa/Vop stimulation, and smaller EPs in the STN during VA stimulation, while the patients were on benzodiazepines.

## 4. Discussion

In contrast to protocols published to date, the procedure reported here (18) allows the simultaneous recording of multiple targets in awake patients without the influence of anesthesia. Therefore, it can be used to describe the effects of BDZ on targeted deep brain regions at rest, and during the electrical stimulation of deep brain nuclei (24). BDZs are not expected to alter brain structure and physical connectivity during the timescale associated with the results reported here. Therefore, changes in DBS EPs are likely to be due to changes in excitability at either the DBS stimulation site, or at the EP site.

In general, the PSDs showed a marked reduction in amplitude when the BDZ were administered for all patients, as the authors conjectured. The widespread distribution of GABA receptors in the pallidum suggested that GPi output would decrease and consequently increase the output of motor thalamus nuclei (Voa/Vop and VA) to the cortex. However, our findings revealed a combined effect on both GPi and thalamus, resulting in the inhibition of both areas. If only GPi were inhibited, the thalamus would have been disinhibited and fired more, whereas if only the thalamus were inhibited, there would have been no change in GPi. Therefore, the combined inhibition of both nuclei is presumably a direct effect of the BDZ, which is consistent with the heterogeneous distribution of GABA receptors (25).

One of the most interesting findings is that the reduction of amplitude in the PSD is the same in the STN and in the GPi. This contrasts with the authors’ expectations, based on the multitude of GABA receptors located in the pallidum (25). Recordings from nonhuman primates (26) and Parkinson’s disease (27) are consistent with the hypothesis of high GPi activity at rest; therefore, the GPi output orthodromic pathway (to thalamus) is reduced by the BDZ, as the results show.

However, if the function of the GPi is potentially altered as has been shown in dystonia (3, 28–30), alternative pathways could be utilized, such as antidromic pathways (e.g., from GPi to STN). In such a case, a stronger signal may be sent to the STN via this antidromic pathway. This is depicted in Figure 6 (green arrow) and provides a plausible explanation for the equal reduction of STN activity despite the pallidum having the largest number of GABA receptors. This hypothesis may help to elucidate the mechanism underlying the effectiveness of DBS in the GPi and STN for treating dystonia.

The results revealed distinct patterns for ON versus OFF benzodiazepines (BDZs) in the context of GPi and VIM stimulation. During GPi stimulation, there was an increase in EP amplitude recorded in the STN, whereas VIM stimulation led to a decrease in EP amplitudes in both the VIM and STN nuclei. These effects may be attributed to the influence of BDZs on thalamic stimulation. The smaller response observed in the VIM during VIM stimulation could potentially impact the EPs elicited in the STN, resulting in a reduction of their amplitudes. This outcome can be explained by the absence of a known direct neurological pathway between the VIM and STN. Therefore, decreased excitability in the source nucleus (VIM) would lead to a lower transmission of signals to other regions, including the STN. Consequently, reduced activity in the VIM during stimulation could result in decreased STN activity as well.

BDZs have widespread effects throughout the brain. However, it is possible that certain regions may experience a comparatively greater level of inhibition, which could influence their sensitivity to electrical stimulation (EPs). Consequently, alterations in both background activity, as demonstrated in the PSDs, and excitability from electrical inputs, which may not all be inhibitory, can occur. These findings suggest a potential shift in the balance between excitation and inhibition across different regions.

In summary, the effects of BDZ on ameliorating hyperkinetic movement disorders are likely multifactorial. The results here suggest that, in some cases, changes in effective connectivity and excitability between deep brain regions could contribute to the BDZ action mechanism. In particular, the authors conjecture that changes in origin or target excitability will lead to changes in the effectiveness of communication between the two regions that could be responsible for changes in the overall pattern of activity, possibly interfering with or attenuating the transmission of the signals responsible for dystonia. Further research is needed to not only confirm this finding in more patients, and but to also determine the relative importance of this effect compared to other mechanisms of BDZ action.

Limitations in the present study include the different underlying diseases and/or areas of brain injury among the patients, as well as the inconsistency of medication types, dosages and timings. The data are also limited by the fact that the recordings are only from electrodes that are placed in potential DBS targets. A full understanding may require recordings from striatum, cortex and brainstem targets; these would help the data interpretation but may not be feasible in humans. Additionally, to obtain more meaningful and dependable results, a larger cohort of patients would be required. Moreover, additional EP measurements such as delay and/or frequency components may also help generate a clearer understanding of the impact of benzodiazepines on EPs. Similar studies in other patient populations would provide insight to understand which, if any, of these trends are unique to dystonic patients, and which others may be a good representation of healthy brain activity.

## Data availability statement

The raw data supporting the conclusions of this article will be made available by the authors, without undue reservation.

## Ethics statement

The studies involving humans were approved by Health Insurance Portability and Accountability Act (HIPAA). The studies were conducted in accordance with the local legislation and institutional requirements. Written informed consent for participation in this study was provided by the participants’ legal guardians/next of kin. Written informed consent was obtained from the individual(s) for the publication of any potentially identifiable images or data included in this article.

## Author contributions

EH-M and TS: conceptualization and methodology, data interpretation, and original draft preparation. JV and JM: project administration and review and editing. EH-M and JV: analyzed the data. JM and TS: conducted the neurological examinations. All authors contributed to the article and approved the submitted version.

## Funding

This work was supported by funding from the Cerebral Palsy Alliance Research Foundation Inc., (PG02518). Research reported in this publication is supported by CHOC.

## Conflict of interest

The authors declare that the research was conducted in the absence of any commercial or financial relationships that could be construed as a potential conflict of interest.

## Publisher’s note

All claims expressed in this article are solely those of the authors and do not necessarily represent those of their affiliated organizations, or those of the publisher, the editors and the reviewers. Any product that may be evaluated in this article, or claim that may be made by its manufacturer, is not guaranteed or endorsed by the publisher.

## References

[1] Sanger TD. Toward a definition of childhood dystonia. Curr Opin Pediatr. (2004) 16:623–7. doi: 10.1097/01.mop.0000142487.90041.a2 15548923

[2] Sanger TD Chen D Delgado MR Gaebler-Spira D Hallett M Mink JW. Definition and classification of negative motor signs in childhood. Pediatrics. (2006) 118:2159–67. doi: 10.1542/peds.2005-3016, PMID: 17079590

[3] Hernandez-Martin E Kasiri M Abe S MacLean J Olaya J Liker M. Globus pallidus internus activity increases during voluntary movement in children with dystonia. iScience. (2023) 26:107066. doi: 10.1016/j.isci.2023.107066, PMID: 37389183 PMC10300218

[4] Jankovic J. Treatment of hyperkinetic movement disorders. Lancet Neurol. (2009) 8:844–56. doi: 10.1016/S1474-4422(09)70183-8 19679276

[5] Stewart K Harvey A Johnston LM. A systematic review of scales to measure dystonia and choreoathetosis in children with dyskinetic cerebral palsy. Dev Med Child Neurol. (2017) 59:786–95. doi: 10.1111/dmcn.13452, PMID: 28485494

[6] Fan W Zhu X Wu L Wu Z Li D Huang F. Propofol: an anesthetic possessing neuroprotective effects. Eur Rev Med Pharmacol Sci. (2015) 19:1520–9. PMID: 25967729

[7] Rudolph U Knoflach F. Beyond classical benzodiazepines: novel therapeutic potential of GABAA receptor subtypes. Nat Rev Drug Discov. (2011) 10:685–97. doi: 10.1038/nrd3502, PMID: 21799515 PMC3375401

[8] Kouvaras E Asprodini EK Asouchidou I Vasilaki A Kilindris T Michaloudis D. Fentanyl treatment reduces GABAergic inhibition in the CA1 area of the hippocampus 24 h after acute exposure to the drug. Neuropharmacology. (2008) 55:1172–82. doi: 10.1016/j.neuropharm.2008.07.025, PMID: 18706433

[9] Kim W Song IH Lim YH Kim MR Kim YE Hwang JH. Influence of propofol and fentanyl on deep brain stimulation of the subthalamic nucleus. J Korean Med Sci. (2014) 29:1278–86. doi: 10.3346/jkms.2014.29.9.1278, PMID: 25246748 PMC4168183

[10] Galvan A Wichmann T. GABAergic circuits in the basal ganglia and movement disorders. Prog Brain Res. (2007) 160:287–312. doi: 10.1016/S0079-6123(06)60017-4 17499121

[11] Kulik Á Nakadate K Nyíri G Notomi T Malitschek B Bettler B. Distinct localization of GABAB receptors relative to synaptic sites in the rat cerebellum and ventrobasal thalamus. Eur J Neurosci. (2002) 15:291–307. doi: 10.1046/j.0953-816x.2001.01855.x 11849296

[12] Sanchez-Vives MV Barbero-Castillo A Perez-Zabalza M Reig R. GABA_B_ receptors: modulation of thalamocortical dynamics and synaptic plasticity. Neuroscience. (2021) 456:131–42. doi: 10.1016/j.neuroscience.2020.03.011, PMID: 32194227

[13] Kerscher C Zimmermann M Graf BM Hansen E. Scalp blocks: a useful technique for neurosurgery, dermatology, plastic surgery and pain therapy. Anaesthesist. (2009) 58:949–60. doi: 10.1007/s00101-009-1604-2 19779756

[14] Berro LF Overton JS Reeves-Darby JA Rowlett JK. Alprazolam-induced EEG spectral power changes in rhesus monkeys: a translational model for the evaluation of the behavioral effects of benzodiazepines. Psychopharmacology. (2021) 238:1373–86. doi: 10.1007/s00213-021-05793-z, PMID: 33594504 PMC8177744

[15] van Lier H Drinkenburg WHIM Van Eeten YJW Coenen AML. Effects of diazepam and zolpidem on EEG beta frequencies are behavior-specific in rats. Neuropharmacology. (2004) 47:163–74. doi: 10.1016/j.neuropharm.2004.03.017, PMID: 15223295

[16] Bastien CH Leblanc M Carrier J Morin CM. Sleep EEG power spectra, insomnia, and chronic use of benzodiazepines. Sleep. (2003) 26:313–7. doi: 10.1093/sleep/26.3.313 12749551

[17] Sanger TD Robison A Arguelles E Ferman D Liker M. Case report: targeting for deep brain stimulation surgery using chronic recording and stimulation in an inpatient neuromodulation monitoring unit, with implantation of electrodes in GPi and Vim in a 7-year-old child with progressive generalized dystonia. J Child Neurol. (2018) 33:776–83. doi: 10.1177/0883073818787741, PMID: 30066598

[18] Sanger T Liker M Arguelles E Deshpande R Maskooki A Ferman D. Pediatric deep brain stimulation using awake recording and stimulation for target selection in an inpatient neuromodulation monitoring unit. Brain Sci. (2018) 8:135. doi: 10.3390/brainsci8070135, PMID: 30018276 PMC6070881

[19] Vitek JL. Pathophysiology of dystonia: a neuronal model. Mov Disord. (2002) 17:S49–62. doi: 10.1002/mds.10142 11948755

[20] Sanger TD Delgado MR Gaebler-Spira D Hallett M Mink JW. Classification and definition of disorders causing hypertonia in childhood. Pediatrics. (2003) 111:e89–97. doi: 10.1542/peds.111.1.e89, PMID: 12509602

[21] Hernandez-Martin E Arguelles E Deshpande R Sanger TD. Evoked potentials during peripheral stimulation confirm electrode location in thalamic subnuclei in children with secondary dystonia. J Child Neurol. (2020) 35:799–807. doi: 10.1177/0883073820931970 32567481

[22] Mallat SG. Multiresolution approximations and wavelet orthonormal bases of *L*^2^(*R*). Trans Am Math Soc. (1989) 315:69–87.

[23] Vidmark J S L Hernandez-Martin E Sanger T D, Increasing consistency of evoked response in thalamic nuclei during repetitive burst stimulation of peripheral nerve in humans. Medical Image Computing and Computer Assisted Intervention—MICCAI 2021, Bruijne M. de, (Springer International Publishing, (2021). 238–247.

[24] Bohn E Goren K Switzer L Falck-Ytter Y Fehlings D. Pharmacological and neurosurgical interventions for individuals with cerebral palsy and dystonia: a systematic review update and meta-analysis. Dev Med Child Neurol. (2021) 63:1038–50. doi: 10.1111/dmcn.14874, PMID: 33772789 PMC8451898

[25] Boyes J Bolam JP. Localization of GABA receptors in the basal ganglia. Prog Brain Res. (2007) 160:229–43. doi: 10.1016/S0079-6123(06)60013-7 17499117

[26] DeLong MR. Primate models of movement disorders of basal ganglia origin. Trends Neurosci. (1990) 13:281–5. doi: 10.1016/0166-2236(90)90110-V, PMID: 1695404

[27] Albin RL Young AB Penney JB. The functional anatomy of basal ganglia disorders. Trends Neurosci. (1989) 12:366–75. doi: 10.1016/0166-2236(89)90074-X 2479133

[28] Schönecker T Gruber D Kivi A Müller B Lobsien E Schneider GH. Postoperative MRI localisation of electrodes and clinical efficacy of pallidal deep brain stimulation in cervical dystonia. J Neurol Neurosurg Psychiatry. (2015) 86:833–9. doi: 10.1136/jnnp-2014-308159, PMID: 25253870

[29] Moll CKE Galindo-Leon E Sharott A Gulberti A Buhmann C Koeppen JA. Asymmetric pallidal neuronal activity in patients with cervical dystonia. Front Syst Neurosci. (2014) 8:15. doi: 10.3389/fnsys.2014.00015 24574981 PMC3920073

[30] Neumann W-J Horn A Ewert S Huebl J Brücke C Slentz C. A localized pallidal physiomarker in cervical dystonia. Ann Neurol. (2017) 82:912–24. doi: 10.1002/ana.25095, PMID: 29130551

