# Supplementary material for: What is the effect of benzodiazepines on deep brain activity? A study in pediatric patients with dystonia
Source: Front Neurol. 2023 Aug 11;14:1215572. doi: 10.3389/fneur.2023.1215572 (PMC10457157; doi:10.3389/fneur.2023.1215572)
Supplement: Supplementary file 1 [file Data_Sheet_1.PDF]

Supplementary material

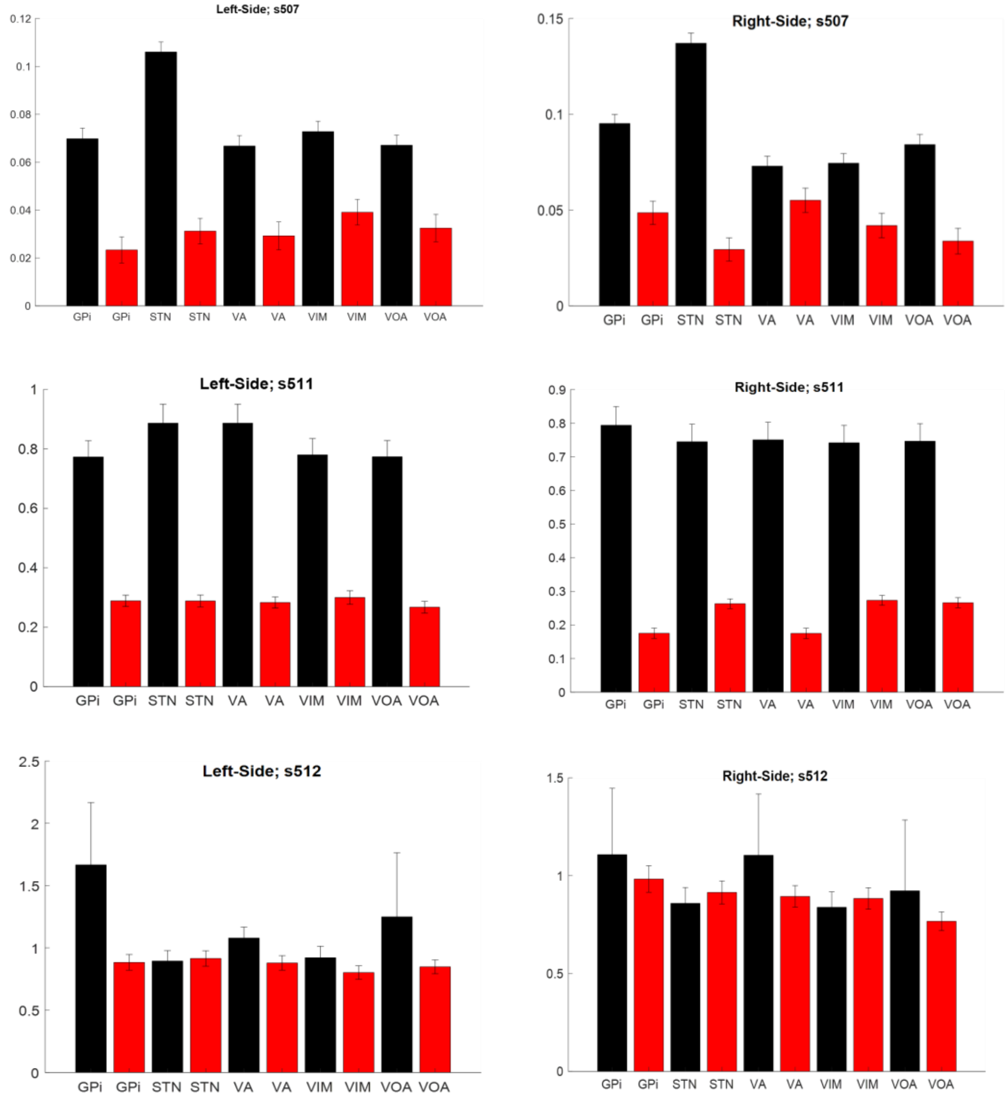

S1. Bar graphics depict the average and standard deviation of the PSD amplitudes between both conditions, benzodiazepine ON (red bars) versus benzodiazepine OFF (black bars), for each brain region from 1 to 50 Hz in each patient. There is significant difference between both conditions ($p<0.05$) for all targeted deep brain for the patients 1 and 2. In the case of patient 3 (s512) shows a significant difference ($p<0.05$) in GPi, VA and VOA. Vertical axis: normalized power differences (dB/Hz). Horizontal axis: targeted deep brain structure.
